# Salvage Nipple-sparing Mastectomy for Patients with Breast Cancer Recurrence: A Case Series of Brazilian Patients

**DOI:** 10.1055/s-0042-1743098

**Published:** 2022-02-24

**Authors:** Antônio Luiz Frasson, Martina Lichtenfels, Fernanda Barbosa, Alessandra Borba Anton de Souza, Ana Beatriz Falcone, Isabela Miranda, Betina Vollbrecht, Carolina Malhone, José Yoshikazu Tariki

**Affiliations:** 1 Hospital Israelita Albert Einstein, São Paulo, SP, Brazil; 2 Pontifícia Universidade Católica do Rio Grande do Sul, Porto Alegre, RS, Brazil

**Keywords:** neoplasm recurrence, subcutaneous mastectomy, segmental mastectomy, neoplasias da mama, mastectomia subcutânea, mastectomia segmental

## Abstract

**Objective** Few studies analyzed the safety of salvage nipple-sparing mastectomy (NSM) for local relapse treatment. We evaluated the outcomes of patients with indications for mastectomy who chose to undergo NSM for ipsilateral breast tumor recurrence (IBTR).

**Methods** Between January 2001 and December 2018, we evaluated 24 women who underwent NSM for local relapse after conservative surgery.

**Results** The patients were followed up for a mean time of 132 months since the first surgery. After the NSM, 5 (20.8%) patients were diagnosed with local recurrence and only 1 (4.2%) patient died. The patients presented 4.8% (2) of partial and 2.4% (1) of total nipple necrosis.

**Conclusion** In this long-term follow-up since the first surgery, we observed low rates of complication and good survival, although associated with high local recurrence in patients diagnosed with IBTR undergoing NSM as salvage surgery. We demonstrated that NSM may be considered after IBTR for patients who did not want to undergo total mastectomy.

## Resumo

**Objetivo** Há poucos estudos sobre a segurança de se realizar adenomastectomia ( *nipple-sparing mastectomy* , NSM, em inglês) para tratamento de recidiva local. O objetivo deste estudo foi avaliar os resultados de pacientes com indicação para mastectomia que optaram por se submeter a NSM para o tratamento de recorrência local.

**Métodos** Foram analisadas 24 pacientes submetidas a NSM para tratamento de recidiva local após tratamento conservador entre janeiro de 2001 e dezembro de 2018.

**Resultados** As pacientes foram acompanhadas por um período médio de 132 meses a partir da primeira cirurgia. Após a NSM, 5 (20,8%) pacientes foram diagnosticadas com recorrência local, e apenas 1 paciente foi a óbito. As pacientes apresentaram 4,8% de necrose parcial e 2,4% de necrose total do mamilo.

**Conclusão** Em um longo período de acompanhamento desde a primeira cirurgia, foram observadas baixas taxas de complicação pós-operatória e boa sobrevida, porém, associadas com uma alta taxa de recorrência local em pacientes submetidas a NSM para tratamento de recidiva local após cirurgia conservadora. Neste estudo, demonstrou-se que a NSM pode ser considerada uma opção cirúrgica para pacientes que não querem se submeter a mastectomia total.

## Introduction

Several factors are related to a higher risk of developing ipsilateral breast tumor recurrence (IBTR), such as high tumor grade, positive excision margins, and younger age. 1 2 Tumor recurrence can be associated to the aggressiveness and progression of the disease. Total mastectomy is the standard surgical treatment for IBTR after breast-conserving surgery (BCS); however, studies have demonstrated that salvage BCS presents oncological safety, and can be used as an alternative to salvage mastectomy in selected patients. 3 4 5

Nipple-sparing mastectomy (NSM) is a conservative mastectomy approach for early breast cancer, with oncological safety and good aesthetic satisfaction. 6 7 The initial indications for NSM excluded patients with previous radiation, ptosis, high body mass index (BMI), and macromastia, although these contraindications have been challenged. Different authors have expanded the classic indications for NSM for patients with previous breast surgery/irradiation, neoadjuvant chemotherapy, and short tumor-nipple distance, and showed safety and low complication rates associated. 8 9 10 11 However, there are few data about the suitability of performing NSM with immediate reconstruction for the treatment of recurrent disease. 12 13 High complication rates were evidenced in patients with a history of previous BCS followed by radiation. 14 15 16 Therefore, the quality of the skin and previous adjuvant radiotherapy (RT) should be considered for reconstruction in this setting.

Aiming to highlight a possible conservative approach, the objective of the present retrospective study is to report our experience with NSM after local recurrence for patients currently indicated for mastectomy, with no involvement of the skin or the nipple-areolar complex (NAC) involvement, who opt to perform NSM.

## Methods

The present retrospective study was performed according to the institutional ethical guidelines, and received approval from the ethics committees of Hospital São Lucas and Hospital Albert Einstein. The study was performed in accordance with The Code of Ethics of the World Medical Association (Declaration of Helsinki). Informed consent was waived by the institutional review board because of the retrospective characteristic of the study.

Patients with IBTR following BCS with an indication for mastectomy with no skin or NAC involvement, and who did not accept NAC resection, were considered to undergo NSM. The risks and benefits from the surgeries were explained to the patients, and they chose to undergo NSM. All patients were operated on by the same surgeon, the data was retrospectively evaluated by the medical chart, and the patients' follow-up was updated during the appointments. Patients with complete data from at least six months follow-up after the salvage surgery were included in the study. From the 348 therapeutic NSMs performed between January 2001 and December 2018, 24 (6.8%) met the inclusion criteria. All patients underwent standard staging examinations, such as radiography or computed tomography (CT) scans of the chest, ultrasound, or CT scans of the abdomen and pelvis, and bone scintigraphy before the salvage surgery. A total of 17 patients underwent bilateral surgeries, 1 patient presented ductal carcinoma in situ (DCIS), 1 patient, invasive ductal carcinoma (IDC) in the contralateral breast, and 15 patients chose contralateral surgery for prophylaxis or symmetrization. All patients had a previous BCS and presented IBTR at the time of the salvage NSM. The data from the previous surgery and previous treatment are listed in Table 1 .

**Table 1:** Previous axillary surgery and systemic treatment in patients undergoing breast-conserving surgery

	N	%
**Previous breast-conserving surgery**	24	100
**Previous axillary surgery**		
Sentinel lymph node biopsy	13	54.2
Axillary lymph node dissection	8	33.3
No axillary surgery	1	4.2
No information	2	8.3
**Number of positive lymph nodes**		
0	19	79.2
1	2	8.3
2	1	4.2
No information	2	8.3
**Previous treatment**		
Hormone therapy	12*	54.6
Chemotherapy	9*	40.9
Radiotherapy	24	100
Anti-human epidermal growth factor receptor-2 (HER2) therapy	2*	9

Note: *Missing data: hormone therapy – *n* = 2; chemotherapy – *n* = 2; and anti-HER2 therapy – *n* = 2.

The patients were followed through clinical examinations at least every six months during the first five years, and yearly thereafter. Other imaging and laboratory tests were left at the discretion of the treating oncologists. Nipple necrosis was defined as any nipple ischemia requiring surgical intervention such as debridement, repair, and skin grafting. The recurrences were diagnosed through clinical examinations and routine imaging tests, and we considered the first relapse presented by the patient as recurrence. All the breast recurrences were biopsied to confirm the tumor. Invasive or in-situ local recurrence was defined as recurrence in the same breast and/or ipsilateral axilla.

All of the procedures were performed under general anesthesia. The NSM skin incision was chosen in accordance with the method of reconstruction and the considerations of the physician. The glandular tissue was removed, leaving only fat tissue to preserve blood supply and reduce the risk of flap necrosis. It is important to highlight that flap thickness varies among patients, since it is based on the amount of subcutaneous fat present in the breast. An intraoperative histopathological examination of frozen sections of the retroareolar tissue was performed to confirm the absence of malignancy in the retroareolar and superficial margins. The margins of glandular tissue dissected were also evaluated by the pathologist. If there were positive margins, a new resection was performed to achieve negative margins. No cutoff point for margin status was used.

**Fig. 1:**
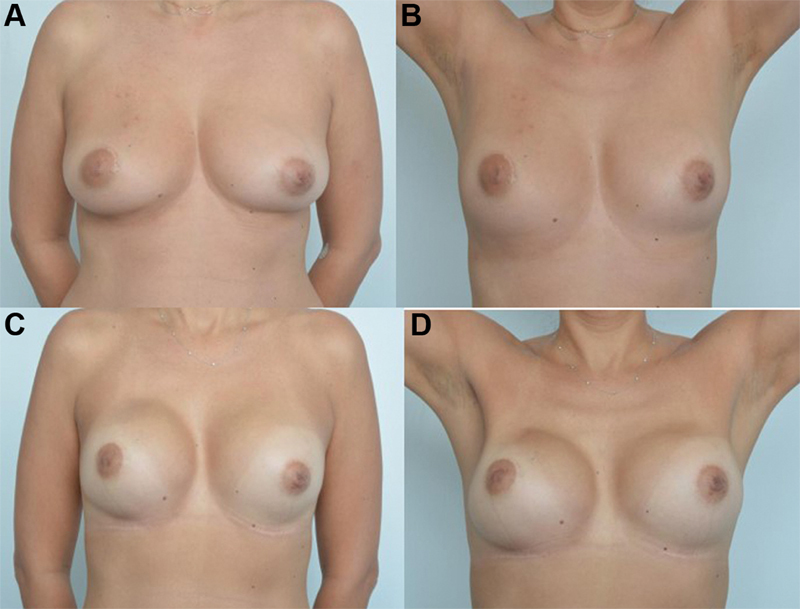
Cosmetic result of the nipple-sparing mastectomy after previous breast-conserving surgery followed by radiotherapy. ( **A,B** ) Preoperative; ( **C,D** ) postoperative: immediate reconstruction with permanent implants.

Sentinel lymph node biopsy (SLNB) was performed when indicated. Immediate breast reconstruction was performed using silicone prosthetic implants or tissue expanders. Inframammary incision was performed in patients who did not need skin excision for ptosis correction. When the patient presented excess skin that needed to be removed, our option was to make a vertical incision between the areola and the inframammary fold. At the end of the reconstruction, we used a mastopexis technique, such as periareolar, vertical, and sometimes transversal scar at the basis of the breast if excessive skin remains. The option with less morbidity and that best replaces the resected breast volume is the use of silicone breast implants. The placement of the implant was through the retromuscular plane in all cases, without the use of matrix ( Fig. 1 ).

Descriptive statistics were used to summarize patient characteristics. The quantitative variables were expressed as means and ranges, while the categorical variables were expressed as absolute and relative frequencies. The rates of disease-free survival (DFS) were summarized using the Kaplan-Meier method and displayed graphically. For a comparison of the median time until the occurrence of the event, the log-rank test was performed. The significance level to claim statistical difference between the groups was set at 0.05. All analyses were performed using the Statistical Analysis System (SAS, SAS Institute Inc., Cary, NC, United States) software, version 9.4.

## Results

Between 2001 and 2018, we observed 71 patients with IBTR after previous BCS. A total of 28 (39.4%) patients underwent repeated BCS, 15 (21.2%) patients underwent total mastectomy, and another 28 (39.4%) patients with an indication for mastectomy with no skin or NAC involvement who chose to undergo NSM. Out of these 28 patients submitted to NSM, 4 were lost to follow-up (< 6 months after the NSM); therefore, we analyzed 24 patients.

The mean duration of the follow-up from the first BCS until the NSM salvage surgery was of 132 months, and the mean time until recurrence after the BCS surgery was of 111 months. In the first surgery, 13 (54.2%) patients underwent SLNB, 8 (33.3%), axillary lymph node dissection (ALND), 1 (4.2%) did not undergo axillary surgery, and data was missing from 2 (8.3%) patients. In total 19 patients presented negative sentinel lymph node (SLN), and 3, positive SLN. All patients were submitted to RT after BCS.

The mean duration of the follow-up after the salvage surgery was of 40 months. The patients' median age at the time of the salvage surgery was 49 years. Overall, 9 (37.5%) patients presented positive familial history of breast cancer, and 1 (4.2%), of ovarian cancer.

Most patients (17; 70.8%) underwent bilateral procedures: 1 (5.9%) patient due to the diagnosis of invasive cancer in both breasts, 1 (5.9%) case of DCIS in both breasts, and 15 (88.2%) patients without disease in the contralateral breast, mostly for prophylactic or esthetic reasons to avoid asymmetry and to achieve a better esthetic result. Frozen sections of the undersurface of the areolar flap of every patient were extracted, and the samples were tumor-free upon analysis. The rates of axillary dissection were low (4.2%), 16 (66.7%) patients underwent SLNB, and the lymph nodes were not evaluated in 7 (29.2%) patients because of previous axillary dissection. All patients were clinically node negative at the time of recurrence. The mean size of the invasive tumor was 2.19 cm, and all tumors were grades 2 or 3. The reconstruction was performed using silicone prosthetic implants for 22 (91.7%) patients, and tissue expander only in 2 (8.3%) cases ( Table 2 ).

**Table 2:** Nipple-sparing mastectomy and systemic treatment

Nipple-sparing mastectomy	n = 24	(%) (100)
*Median age in years (range)*	49 (36–78)	−
*Menopausal status*		
Premenopausal	12	50
Postmenopausal	12	50
*Unilateral surgery*	7	29.2
*Bilateral surgery*	17	70.8
In-situ contralateral breast cancer	1	5.9
Invasive contralateral breast cancer	1	5.9
Prophylactic contralateral nipple-sparing mastectomy	15	88.2
*Axillary surgery*		
Sentinel lymph node biopsy	16	66.7
Axillary lymph node dissection	1	4.2
No surgery	7	29.2
*Mean lesion size (cm)*	2.19	
*Histology*		
Invasive ductal carcinoma	14	58.4
Invasive lobular carcinoma	5	20.8
Ductal carcinoima in situ	5	20.8
*Grade*		
1	0	0
2	12	63.2
3	7	36.8
*Molecular subtype*		
ER +/PR +/HER2-	11	58
ER +/PR +/HER2+	4	21
ER-/PR-/HER2+	2	10.5
ER-/PR-/HER2-	2	10.5
*Systemic treatment*		
Hormone therapy		
Tamoxifen	5	20.8
Aromatase inhibitor	9	37.5
No	10	41.7
Unknown		
Chemotherapy		
Neoadjuvant	2	8.3
Adjuvant	9	37.5
No	13	54.2
Unknown		
Anti-HER2 therapy		
Trastuzumab	3	12.5
Double block	3	12.5
No	18	75
Unknown		
Radiotherapy		
Yes	6	25
No	18	75
Unknown		

Abbreviations: ER, estrogen receptor; HER2, human epidermal growth factor receptor-2; PR, progesterone receptor. Table 3 Complication rates and recurrence after nipple-sparing mastectomy

Out of the 41 NSMs performed, 4 (9.6%) complications occurred, including 1 (2.4%) infection, 2 (4.8%) cases of partial nipple necrosis, and 1 (2.4%) case of total nipple necrosis ( Table 3 ). The patient with total nipple necrosis was treated through the conservative approach, and recovered completely, maintaining the nipple.

**Table 3:** Complication rates and recurrence after nipple-sparing mastectomy

Complications	n = 41*	%
Infection	1	2.4
Partial nipple necrosis	2	4.8
Total nipple necrosis	1	2.4
**Recurrences**	**n = 24**	**%**
Local recurrence	5	20.9
Same breast/same quadrant	1	−
Same breast/other quadrant	3	−
Nipple-areolar complex	1	−
Distant metastasis	1	4.1
New primary tumor		1 4.1

Note: * The complications were calculated by the number of procedures performed ( *n* = 41).

During the mean follow-up of 40 months (range: 6 to 156 months) after the NSM, 5 (20.9%) patients were diagnosed with local recurrence, being 1 (4.1%) in the NAC. None of the patients presenting local relapse underwent reirradiation. Bone metastasis was observed in 1 (4.1%) patient, and 1 (4.1%) patient developed a new primary kidney tumor. The relapses are shown in Table 3 . The characteristics of the local recurrences are shown in Table 4 .

**Table 4:** Characteristics of local recurrence after nipple-sparing mastectomy

		Second recurrence – after NSM				
Time until the first recurrence after BCS (months)	Time until the second recurrence after NSM (months)	Age (years)	Location of the recurrence	New surgery	Pathology	Survival
12	9	41	Same breast/other quadrant	Total mastectomy	IDC: bifocal; 4.5 cm and 0.9 cm; grade 3; ER-/PR-/HER2+	Alive
27	6	59	Same breast/other quadrant	Total mastectomy	DCIS: 2.8 cm; multifocal; grade 3	Alive
55	41	50	Same breast/other quadrant	Total mastectomy	IDC: 0.9 cm and 1.4 cm; bifocal; grade 3; ER +/PR +/HER2+	Alive
75	32	48	NAC	BCS	DCIS: multifocal; grade 3	Alive
117	83	50	Same breast/same quadrant	Total mastectomy	DCIS: multifocal; grade 2	Alive

Abbreviations: BCS, breast-conserving surgery; DCIS, ductal carcinoma in situ; ER, estrogen receptor; HER2, human epidermal growth factor receptor-2; PR, progesterone receptor; IDC, invasive ductal carcinoma; NAC, nipple-areolar complex; NSM, nipple-sparing mastectomy; PR, progesterone receptor.

**Fig. 2:**
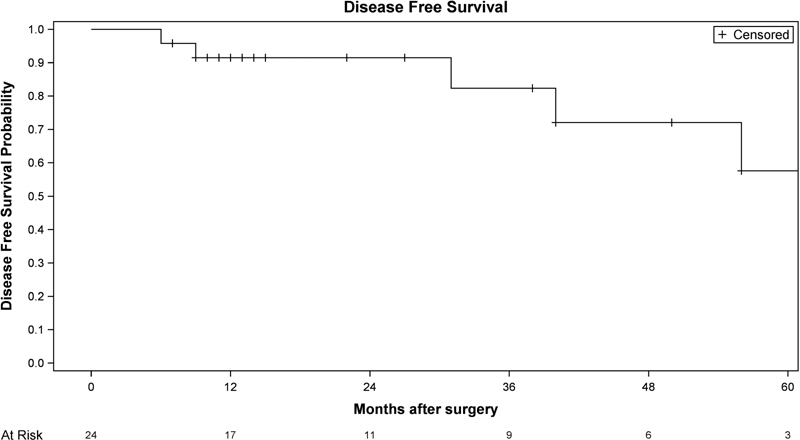
Disease-free survival of breast cancer patients.

The overall survival rate was of 95.8%, and 1 patient died from progression of the breast cancer. Figure 2 shows the rates of DFS, which was defined as the time from the performance of the NSM until invasive or in situ, local, regional, or distant recurrence, the development of a second primary tumor, or death from any cause. Overall survival was defined as the time from the performance of BCS to death from any cause.

## Discussion

The suitability of NSM for the treatment of recurrent disease after BCS and whole-breast radiation was evidenced at a short-term follow-up with low complication rates, successful preservation of the NAC and satisfactory oncological outcomes. 13 However, the safety of performing immediate reconstruction in the salvage surgery is still under investigation, and few studies have been published. The present is the first study to show the outcomes of a case series of Brazilian patients diagnosed with IBTR after a previous conservative surgery, who underwent NSM with immediate reconstruction as a salvage surgery ( Chart 1 ).

**Chart 1:** Previous studies on NSM for local recurrence after BCS

Authors (year)	Number of patients	Complication rates	Recurrence rates	Survival	Follow-up (months)
Cordeiro et al. (2012) 14	121 patients who underwent NSM after recurrence + RT; 1.578 patients who underwent NSM for early cancer	Flap necrosis: 18% x 7.7%; infection: 8.3% x 4.8%; hematoma: 0.8% x 1.8%	NA	NA	NA
Lam et al. (2015) 15	22	Seroma: 13.6%; bleeding: 45.%; delayed wound healing: 4.5%; infection: 4.5%	LR: 2	NA	NA
Murphy et al. (2017) 13	21	Postoperative, requiring intervention: 2; flap necrosis: 1; seroma: 2	0%	100%	Median: 14.6
Lee et al. (2019) 12	18 patients who underwent NSM after recurrence; 127 patients who underwent NSM for early cancer	Infecion: 5.6% x 3.9%; nipple necrosis: 11.1% x 17.3%	LR: 5.6% x 3.1% Metastasis: 0% x 5.5% Contralateral BC: 5.6% x 0.8%	100% x 97.6%	Mean: 45.5 × 45.3

Abbreviations: BCS, breast-conserving surgery; NA, not applicable; NSM, nipple-sparing mastectomy; RT, radiotherapy; BC, breast cancer; LR, local recurrence.

Some tumor characteristics, such as size and molecular subtype, were lost due to the long-term follow-up since the first surgery; therefore, this data was not evaluated. After the first surgery, the mean time until recurrence was of 111 months. All patients presented IBTR and had an indication for mastectomy; however, they presented no skin or NAC involvement, and chose to undergo salvage NSM. The patients were informed about the risks and benefits of both surgeries, and they decided not to undergo mastectomy. Most patients were young, half of them were premenopausal, and presented large and high-grade invasive tumors. Their decision to undergo NSM patients might be related to the young age and the esthetic outcomes related to the surgery. 17 Galimberti et al., 18 in their study, found that 72.1% of the patients undergoing NSM aged < 50 years, and 77.1% were premenopausal. The maintenance of the NAC confers a significant improvement in sexual, physical and psychosocial satisfaction in breast cancer patients undergoing NSM compared with skin-sparing mastectomy (SSM). 19

Many patients underwent bilateral surgeries, and almost 90% of them did not have a diagnosis of cancer in the contralateral breast, and chose the surgery for prophylaxis or esthetic reasons, basically symmetry. Women choose to undergo the prophylactic procedure aiming to optimize the oncologic outcome, despite the fact that the literature shows no benefit in terms of survival. 20 21 Contralateral mastectomy is associated with young age, significant family history of breast or ovarian cancer, high level of schooling, and great worry about recurrence, the same characteristics we have observed in our patients who decided to undergo contralateral surgery. 22

The number of patients with no lymph node assessment was high (29.2%) in the salvage surgery; however, all of them were submitted to a previous axillary surgery in the first surgical procedure, and the preoperative images did not evidence suspicious lymph nodes. Patients with previous breast surgery and complete axillary dissection do not need further axillary surgery. 23 The patients previously submitted to SLNB during the first surgery who presented clinically-negative axilla at the time of recurrence underwent a new SLNB (66.7%).

Our rate of complications was low (9.6%), and in line previous studies 24 that showed no increase in postoperative complications in patients undergoing NSM after a previous BCS. Murphy et al. 13 evaluated 19 patients who underwent NSM for the treatment of recurrent breast cancer, and observed complications requiring intervention in two patients, one with flap necrosis and one with seroma. Despite the retrospective nature of the present study, the complications and relapses from the NSM performed by our team were well documented in the medical charts.

Most of our patients were submitted to reconstruction with silicone implants (91.7%) and only 2 (9%) presented capsular contracture that caused breast asymmetry. These patients underwent a new surgery with capsular resection and a silicone prothesis exchange.

We observed 5 (20.8%) patients diagnosed with local recurrence in a mean follow-up of 40 months after the salvage surgery. Murphy et al. 13 did not observed any cases of cancer recurrence in patients undergoing NSM for the treatment of IBTR; however, their follow-up was shorter compared with the present study, with a median of 14.6 months. 13 Lee et al. 12 evaluated the oncological safety and survival of 18 patients who underwent NSM and immediate reconstruction for recurrence after BCS, and compared them with 127 patients who underwent NSM as the primary treatment for breast cancer. The patients undergoing a secondary NSM had mean age of 45 years, the majority was diagnosed with IDC (61.1%), none presented invasive lobular carcinoma (ILC), 53.8% presented stage-I tumors, 30.8% Tis, and no grade-3 tumors. Most of the patients (77.8%) in the secondary NSM group underwent preoperative radiotherapy. The authors 12 found similar rates of surgical complications and outcomes between the two groups during a mean follow-up of 45 months. The rates of local recurrence was of 5.6% in the secondary NSM group, against 3.1% in the primary NSM group. At the end of the follow-up, all patients in the secondary group and 97.6% in the primary group were alive. 12 The rate of local recurrence found in this study 12 was lower than the one observed in the present study; however, in our cohort there were more cases of ILC (21%) and high-grade tumors (graded 2 or 3), which are associated with an increased risk of relapse. Tumor grade is one of the parameters used to determine survival in all cases of breast cancer, and a high histologic grade has been associated with a high recurrence score on oncotype Dx. 25 The ILC histology is also related to higher risk of recurrence, with worse outcomes after 5 years and disease-specific survival compared with IDC. 26

In the CALOR trial, 27 the authors evaluated the effectiveness of adding chemotherapy after surgery to treat isolated locoregional recurrence (ILRR). In the study, 27 40% of the included patients underwent a previous mastectomy, and mosts presented ER-positive ILRR. In a 10-year follow-up, the authors observed a rate of DFS of 70% among patients who underwent chemotherapy, and of 34% among patients who did not. However, for patients with ER-positive tumors, the chemotherapy presented no benefits. The overall survival was of 73% in ER-negative ILRRs, and of 76% in ER-positive cases who underwent chemotherapy. 27 In the present study, the survival rate was high: only one patient died from disease progression in a long-term follow-up since the first surgery. Compared with the CALOR trial, 27 which included patients with previous mastectomy, our patients presented a better prognostic, since all of them underwent previous BCS followed by RT. It is expected that patients with ER-positive tumors present delayed recurrence and death after treatment. In the present study, the mean duration of the follow-up after the salvage surgery was of 40 months, and this could have influenced the high survival rates found among our population.

The present study has limitations, including the retrospective design of the analysis, the small sample size, and the process of selection of patients, which did not include thoses with incomplete data and those submitted to surgeries other than BCS as the first treatment. We did not compare the results of the NSM with those of the repeated BCS or total mastectomy, the standard treatment, because of the small sample size. Our aim was to highlight the possibility of performing NSM to treat recurrence in patients who did not want to undergo mastectomy and had no indication for BCS. Future studies comparing the outcomes of the different surgeries to treat IBTR are needed.

## Conclusion

In a mean follow-up of 40 months after NSM, we observed ∼ 20% local recurrence after the conservative approach in the management of local relapse in a case series of Brazilian patients. We also highlight a high survival rate in patients diagnosed with IBTR after a previous conservative surgery who underwent NSM as salvage procedure in a long-term follow-up since the first surgery. The complication rates were low. Therefore, we suggest that NSM may be performed after IBTR for patients with an indication for mastectomy with no skin or NAC involvement, who do not wish to undergo mastectomy to avoid a mutilating surgery.
